# Comparing Peak Burn Injury Times and Characteristics in Australia and New Zealand

**DOI:** 10.3390/ijerph19159578

**Published:** 2022-08-04

**Authors:** Rebecca Hong, Monica Perkins, Belinda J. Gabbe, Lincoln M. Tracy

**Affiliations:** 1 School of Public Health and Preventive Medicine, Monash University, Melbourne, VIC 3004, Australia; 2 Health Data Research UK, Swansea University Medical School, Swansea University, Swansea SA2 8PP, UK

**Keywords:** burns, flame, scald, cooking, registry

## Abstract

Burns are a leading cause of morbidity and mortality worldwide. Understanding when and how burns occur, as well as the differences between countries, would aid prevention efforts. A review of burn injuries occurring between July 2009 and June 2021 was undertaken using data from the Burns Registry of Australia and New Zealand. Peak injury times were identified on a country-by-country basis. Variations in demographic and injury event profiles between countries were compared using descriptive statistics. There were 26,925 admissions recorded across the two countries (23,323 for Australia; 3602 for New Zealand). The greatest number of injuries occurred between 6 PM to 7 PM in Australia (1871, 8.0%) and between 5 PM to 6 PM in New Zealand (280, 7.8%). In both countries, scalds accounted for the greatest proportion of injuries during peak times (988, 45.8%), but a greater proportion of young children (under three years) sustained burns during New Zealand’s peak times. The number of burn injuries associated with the preparation and/or consumption of food offers an opportunity for a targeted prevention program that may yield benefits across the two countries. Age- and mechanism-related differences in the profile of burn-injured patients need to be considered when developing and implementing such a program.

## 1. Introduction

Burn injuries are a complex and unique form of trauma affecting people of all ages from all countries. As part of the 2016 Global Health Estimate, the World Health Organization (WHO) attributed >150,000 deaths annually to burns [1]. However, the true impact of burns extends beyond the number of deaths. In 2017, burn injuries accounted for >8.4 million disability-adjusted life years [2] highlighting the significant medical, social, economic, and personal burden associated with these injuries [3,4,5]. For example, physical disfigurement from burn scar contractures can impact psychological health, cause social stigma, and lead to social isolation and abandonment [6]. The burden of burn injuries is not distributed evenly across the globe, with higher rates of morbidity and mortality observed across low- and middle-income countries when compared with high-income countries [7]. However, this should not mean that prevention initiatives should wholly focus on low- and middle-income countries, as the vast majority of burn injuries can be prevented through effectively designed systems and initiatives [6].

Two crucial parts of any injury prevention approach are surveillance and analysis. Effective prevention interventions cannot be designed without a concise description of the problem at hand [6]. International research has demonstrated certain times of day where burn injury figures appear to rise and fall considerably. Data from Australia and New Zealand show a peak in burns occurring between 11 PM and midnight, with 13% of all burns falling within this period [8]. In contrast, the United Arab Emirates revealed peaks at midday and again at 4 PM [9], while data from Taiwan demonstrated a spike in burns occurring between 4–6 PM and another peak from 10 PM to midnight [10]. Celko and colleagues reported that children in the Czech Republic sustained a greater number of burn injuries at a specific time of day and that this could be further differentiated depending on the place of injury. For example, burns occurring inside the home were found to peak at 11 AM and 7 PM, whereas burn injuries occurring outside the home peaked at 4 PM [11]. These location-specific peaks present potential prevention opportunities. However, previous studies have often only included data from a single hospital, meaning the results may not be generalised within their own country or in other countries. A more informative, international perspective is required, as this may identify similarities in peak times and injury mechanisms across countries where similar prevention initiatives may be utilised.

Although peaks in burn injury times in Australia and New Zealand have been explored in informal reports, there has yet to be a detailed examination of the types of patients who sustain their injuries during these peak times, nor have the types of injuries sustained been considered. Therefore, the aim of this study was to identify the peak times when burn injuries occurred in Australia and New Zealand, as well as describe the demographic and injury event characteristics of affected patient populations. Understanding the association between when and how burn injuries occur in different geographical settings is important to inform targeted prevention strategies for different countries.

## 2. Materials and Methods

### 2.1. Data Source

This study used data from the Burns Registry of Australia and New Zealand (BRANZ) for injuries occurring between July 2009 and June 2021. The BRANZ has collected epidemiological, quality of care, and in-hospital outcome data for paediatric and adult burn patients across Australian and New Zealand specialist burn services since July 2009. Additional details about the BRANZ, such as inclusion and exclusion criteria, have been published elsewhere [12,13,14]. In brief, patients are included in the BRANZ if they are admitted to an Australian and New Zealand burn service within 28 days of injury and (a) the admission lasts for more than 24 h, or (b) the admission lasts for less than 24 h but the patient undergoes a burn wound management procedure in theatre. All in-hospital deaths are included regardless of admission duration. All patients meeting BRANZ inclusion criteria were included in the study. Patients or the public were not involved in the design, reporting, or dissemination of this research.

### 2.2. Data Management

The date and time of injury are recorded by the BRANZ. Within the BRANZ, midnight is entered as either 23:59 the preceding day or as 00:01 the following day (00:00 and 24:00 are not accepted as valid entries). Patients with missing or unknown time of injury data (recorded as 00:00 in BRANZ) were excluded from analysis. The day of the week the injury occurred was extracted from date of injury data. Time of injury data was grouped according to the hour the injury occurred in. The hour period(s) when the greatest number of injuries occurred were deemed the “peak” injury time.

Patients were classified as paediatrics if they were <16 years at the time of injury, adults if they were 16–64 years at the time of injury, and older adults if they were ≥65 years at the time of injury [15]. Age at the time of injury was also categorised as per BRANZ Annual Reports: 0–12 months, 13–24 months, 25–36 months, 3–5 years, 6–10 years, 11–15 years, 16–19 years, 20–29 years, 30–39 years, 40–49 years, 50–59 years, 60–69 years, 70–79 years, and ≥80 years [8]. A binary variable for male gender was coded (male = 1; female or intersex/indeterminate = 0).

The primary cause of the burn injury was categorized as scald, flame, contact, or other cause (including, but not limited to, electrical, chemical, and friction burns). The most common injury subcauses, (e.g., scald from tap water, etc.), were identified. The percentage of total body surface area (TBSA) burned was reported as a continuous variable. The maximal recorded depth of the burn was categorised as superficial, mid-dermal, deep dermal, or full thickness. Associated inhalation injury was coded as a binary variable (yes = 1, no = 0). Place of injury was coded as home or usual residence or another place. Injury intent was coded as unintentional or other known intent. The need for burn wound management in theatre during the admission was coded as a binary variable (yes = 1, no = 0), as was the presence or absence of an inhalation injury. Hospital length of stay (LOS) was calculated from date and time of admission and discharge data. Discharge status was recoded to home or usual residence or other disposition.

### 2.3. Statistical Analysis

Summary statistics were used to describe the data. Frequencies and percentages were used for categorical variables. Medians and interquartile ranges (IQR) were used for continuous variables, due to the skewed nature of the data. Data were presented by country and by peak injury time status. Statistical analyses were not undertaken due to the exploratory and descriptive nature of the study. Data were managed using Stata 17.0 (StataCorp, College Station, TX, USA). Figures were produced in the R statistical environment version 4.1.3 (R Development Core Team, Vienna, Austria) [16] using the *plyr* [17], *tidyverse* [18], *RColorBrewer* [19], and *scales* [20] packages.

## 3. Results

### 3.1. Demographic and Injury Characteristics

Data for 26,925 admissions (23,323 for Australia; 3602 for New Zealand) were included in the study (Figure S1). The demographic and injury characteristics, presented for the overall sample and by country, are summarized in Table 1. Two-thirds of admissions were male, and the median (IQR) age was 27 (8–47) years. Scalds and flame burns were the predominant causes of injury, accounting for more than three-quarters of admissions. More than half the admissions had a deep dermal or full-thickness burn, but only a small proportion had an inhalation injury in addition to their cutaneous burn. Almost two-thirds of injuries occurred in the home (or usual place of residence); almost all burns were unintentional. Three-quarters of admissions underwent a burn wound management procedure in theatre. The median (IQR) hospital LOS was 4.0 (1.6–9.8) days. More than 80% of admissions were discharged to their home or usual residence.

### 3.2. Peak Injury Times and Trends

**Figure 1:**
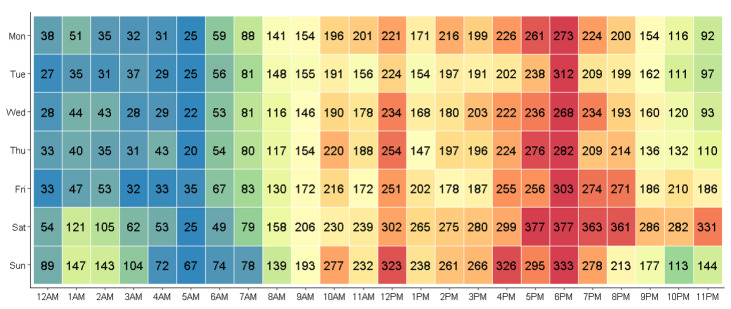
Case distribution by day and time of injury.

The greatest number of burn injuries occurred on Saturday (*n* = 5179, 19.2%; Figure 1), followed by Sunday (*n* = 4582, 17.0%) and Friday (*n* = 3832, 14.2%). The greatest number of burn injuries occurred from 6 PM to 7 PM (*n* = 2148, 8.0%), 5 PM to 6 PM (*n* = 1939, 7.2%) and 12 PM to 1 PM (*n* = 1809, 6.7%). When considered individually, the peak injury time for Australia was from 6 PM to 7 PM (*n* = 1871, 8.0%) and from 5 PM to 6 PM in New Zealand (*n* = 280, 7.8%; Figure S1). The largest proportion of injuries were reported to occur on the hour (*n* = 15,091, 56.1%), on the half-hour (*n* = 6922, 25.7%), or at a quarter to the hour (*n* = 1141, 4.2%).

**Figure 2:**
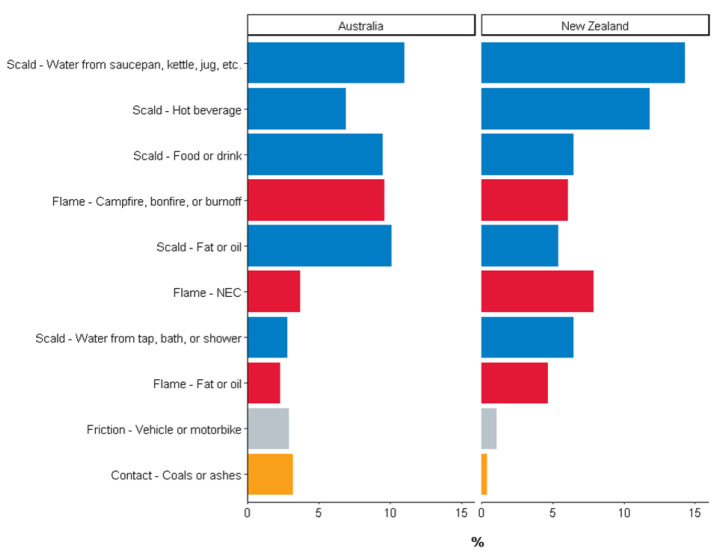
Top 10 injury subcauses during peak injury time by country. NEC = not elsewhere classified.

Characteristics of burn injuries occurring during peak times for each country are summarized in Table 2. Australian patients injured during the peak injury time had a median (IQR) age of 27 (6–48) years, while New Zealand patients had a median (IQR) age of 18 (1–40) years. A smaller proportion of adults and older were injured in New Zealand peak times, while a greater proportion of paediatric patients were injured during peak times (Figure S2). In both countries, scalds accounted for the greatest proportion of injuries during peak times, resulting from water spilt from a saucepan, kettle, jug, etc., a hot beverage, fat or oil, or a tap, bath, or shower (Figure 2). New Zealand peak time burns affected a greater median TBSA but was often not as deep compared to Australian peak time burns. Inhalation injury was rare. More than three-quarters of burns during peak injury times occurred in the home, while more than 90% of burns were unintentional. Over 70% of patients underwent a burn wound management procedure in theatre. Patients injured during peak times spent a median of 4–5 days in hospital, but almost 90% were discharged to their home or usual residence.

## 4. Discussion

Burns cross the social–cultural–economic divides of the world, with no country or people exempt from burn injury. The universality of burns offers a unique opportunity for analyses to capture and explore *when* and *how* burns occur on an international scale. A binational clinical quality registry receiving data from all specialist burn services across two countries was used to identify when there are peaks in burn injuries throughout the day, how these injuries occur, and whom they affect. Although our findings reveal a slight difference in the peak time when the injury occurred and the characteristics of affected patients, certain similarities were observed.

Burns during peak injury times in the current study were closely associated with cooking: scalds from hot water in saucepans, scalds from foods (both liquid and solid) and hot beverages, and scalds from fat or oil. This is consistent with the epidemiological analysis of burns in low- and middle-income countries from 2006, which shows that regardless of age, most burn injuries occurred in the kitchen and were related to food being prepared, cooked, or served [21]. Other studies have also found similar links between meal preparation and meal times associated with certain peaks in burn occurrences throughout the day [9,10,11]. Although scalds were the most common cause of injury in the current study, 36% of patients (both in the overall sample and in patients who sustained their injury during peak times) sustained a flame burn. This finding is somewhat expected, as adults account for the majority of data within the BRANZ, and flame burns are the most common cause of injury in adult patients aged 16–64 [8]. Nevertheless, the findings of the current study reinforce the importance of campfire/bonfire safety, and the need to take the utmost caution when cooking to prevent flame-related oil burns.

A key finding of the current study was the age-related differences observed in patients injured in Australia and New Zealand during peak injury times. Forty-eight percent of New Zealand patients injured during peak injury times were paediatric patients, compared to 36% of Australian patients injured during peak times. Specifically, a greater proportion of New Zealand peak time injuries occurred in children under the age of three. Age-related differences in burn injury prevalence and mechanism between Australia and New Zealand have previously been reported [22]. As scalds from saucepans, kettles, and hot beverages were the most common injury subcauses during peak times in New Zealand, the current findings may arise through a larger number of New Zealand children reaching up to tables or benchtops and pulling saucepans or cups containing hot liquid onto themselves. An app-based intervention aimed at improving knowledge about the risks of hot beverage scalds in young children has proven to be effective in a Queensland-based population [23]. Trialling such an intervention in New Zealand may lead to similar knowledge improvement and a subsequent reduction in paediatric scalds.

Research into the prevalence and prevention of cooking-related burns has largely focused on lower- and middle-income countries. Reasons as to why susceptibility to burns occur in the household during mealtimes may be due to a number of factors such as living environments where living spaces for kitchen and dining areas are not separated, overcrowding, and specific methods of cooking used in a particular country. For example in Taiwan, small dishes are cooked at the dining table using a small charcoal grill [24]. A study of risk factors for paediatric burns in Mongolia found that children aged zero to three years were at an increased risk of scalds in Gers, a traditional tent-like house, with only a single living space. This posed a risk during food preparation because electric pots were commonly placed on the floor where children were able to fall into them [25]. Additionally, house designs have been highlighted as a risk factor for burns in earlier studies. In the Czech Republic, prefabricated apartments do not include a dining room [11], and in Hong Kong, hot soups and kettles are often taken into the living room to cool down due to the lack of space in kitchens [26]. These findings have led to unique preventative strategies adapted to suit regional risk factors. For instance, in Mongolia, a kitchen rack has been developed as a burns prevention tool for children [27], and in Sri Lanka, the implementation of the Safe Bottle Lamp program in the 1990s addressed the unsafe homemade kerosene lamps commonly used within homes [28]. Such strategies may encourage additional prevention initiatives in high-income countries such as Australia and New Zealand.

This study has limitations that must be considered when interpreting the findings. The BRANZ only collects data on burn-injured patients who present to specific hospitals with their injuries meeting additional criteria relating to injury severity [12]. Therefore, the data presented in this paper underestimates the complete number of burn injuries sustained across Australia and New Zealand. Additionally, this project identified a coding error in previous reports from the BRANZ that misrepresented peak injury times, meaning caution must be taken when considering data in older reports [8]. Finally, this study only compared data from two high-income countries, meaning the results may not translate to other (low-income) countries. The WHO Global Burn Registry (GBR) was established in 2018 to serve as a centralised collection of burn injury data from low- and middle-income countries and has yielded several papers examining different aspects of burn care [7,29,30,31,32]. However, the voluntary nature of enrolment for the WHO GBR means data captured within and between the countries that utilise this registry may be incomplete. For example, not all hospitals within each country may contribute data to the GBR. In addition, such a study cannot be performed using GBR data as unknown or missing data are entered as midday [33], meaning there is no way to confirm which injuries truly occurred at midday and which occurred at unknown times. Other data sources may be required to replicate the current study in other countries.

## 5. Conclusions

Although peak burn injury times varied slightly between Australia and New Zealand, there was a strong link between the timing of burns and cooking. Burns occurring within the household were predominantly flames or scalds, with paediatric populations primarily affected by scalds. Given the country-specific variation in the age groups of patients affected by burns during these peak times, there may be opportunities for prevention programs to be tailored to reflect these differences.

## Figures and Tables

**Table 1:** Demographic and injury characteristics of overall sample.

	BRANZ (*n* = 26,925)	Australia (*n* = 23,323)	New Zealand (*n* = 3602)
Age, median (IQR) years ^a^	27 (8–47)	28 (11–47)	21 (2–42)
Age group ^a^			
Paediatric (< 16 years)	8323 (30.9%)	6771 (29.0%)	1552 (43.1%)
Adult (16–64 years)	16,227 (60.5%)	14,456 (62.0%)	1821 (50.6%)
Older adult (≥ 65 years)	2321 (8.6%)	2092 (9.0%)	229 (6.4%)
Gender ^b^			
Male	18,378 (68.3%)	15,937 (68.4%)	2441 (67.8%)
Other	8537 (31.7%)	7379 (31.6%)	1158 (32.2%)
Primary cause ^c^			
Scald	9912 (36.9%)	8244 (35.4%)	1668 (46.4%)
Flame	9660 (35.9%)	8413 (36.1%)	1247 (34.7%)
Contact	4012 (14.9%)	3604 (15.5%)	408 (11.3%)
Other cause	3309 (12.3%)	3034 (13.0%)	275 (7.6%)
TBSA, median (IQR) % ^d^	3.0 (1.0–7.6)	3.0 (1.0–7.0)	5.0 (2.0–9.0)
Maximal depth recorded ^e^			
Superficial dermal	3633 (15.2%)	2832 (13.5%)	801 (26.8%)
Mid-dermal	6636 (27.7%)	5719 (27.3%)	917 (30.7%)
Deep dermal	8138 (34.0%)	7621 (36.4%)	517 (17.3%)
Full thickness	5514 (23.1%)	4760 (22.7%)	754 (25.2%)
Inhalation injury ^f^			
No	25,512 (95.0%)	22153 (95.2%)	3359 (93.8%)
Yes	1341 (5.0%)	1118 (4.8%)	223 (6.2%)
Place of injury ^g^			
Home/usual residence	16,541 (63.1%)	14,047 (61.6%)	2494 (73.3%)
Other place	9657 (36.9%)	8750 (38.4%)	907 (26.7%)
Injury intent ^h^			
Unintentional injury	25,365 (94.5%)	21,965 (94.5%)	3400 (94.7%)
Other intent	1479 (5.5%)	1289 (5.5%)	190 (5.3%)
Burn wound management in theatre ^i^			
No	6902 (25.8%)	6120 (26.4%)	782 (22.0%)
Yes	19,835 (74.2%)	17,056 (73.6%)	2779 (78.0%)
LOS, median (IQR) days ^j^	4.0 (1.6–9.8)	4.0 (1.4–9.8)	3.8 (2.0–9.8)
Discharge disposition ^k^			
Home/usual residence	23,291 (86.7%)	20,109 (86.4%)	3182 (88.5%)
Other disposition	3585 (13.3%)	3173 (13.6%)	412 (11.5%)

Data presented as frequency (percentage) unless otherwise specified. BRANZ = Burns Registry of Australia and New Zealand; IQR = interquartile range; LOS = length of stay; TBSA = total body surface area. Data missing for ^a^ 4 admissions, ^b^ 10 admissions, ^c^ 32 admissions, ^d^ 614 admissions, ^e^ 349 admissions, ^f^ 72 admissions, ^g^ 727 admissions, ^h^ 81 admissions, ^i^ 188 admissions, ^j^ 17 admissions, and ^k^ 49 admissions.

**Table 2:** Demographic and injury characteristics of patients injured in peak times.

	Non-Peak (*n* = 24,474)	Australia (6 PM; *n* = 1871)	New Zealand (5 PM; *n* = 280)
Age, median (IQR) years ^a^	27.0 (9.0, 47.0)	27.0 (6.0, 48.0)	18.0 (1.0, 40.0)
Age group ^a^			
Paediatric (< 16 years)	7523 (30.4%)	666 (35.6%)	134 (47.9%)
Adult (16–64 years)	15,127 (61.0%)	1019 (54.5%)	131 (46.8%)
Older adult (≥ 65 years)	2120 (8.6%)	186 (9.9%)	15 (5.4%)
Gender ^b^			
Male	16,999 (68.6%)	1204 (64.4%)	175 (62.5%)
Other	7767 (31.4%)	665 (35.6%)	105 (37.5%)
Primary cause ^c^			
Scald	8924 (36.1%)	845 (45.2%)	143 (51.1%)
Flame	8972 (36.3%)	591 (31.6%)	97 (34.6%)
Contact	3726 (15.1%)	258 (13.8%)	28 (10.0%)
Other cause	3122 (12.6%)	175 (9.4%)	12 (4.3%)
TBSA, median (IQR) % ^d^	3.0 (1.0, 7.7)	3.0 (1.0, 7.0)	5.5 (2.5, 10.0)
Maximal depth recorded ^e^			
Superficial dermal	3340 (15.2%)	243 (14.6%)	50 (21.8%)
Mid-dermal	6116 (27.8%)	439 (26.3%)	81 (35.4%)
Deep dermal	7417 (33.7%)	670 (40.1%)	51 (22.3%)
Full thickness	5150 (23.4%)	317 (19.0%)	47 (20.5%)
Inhalation injury ^f^			
No	23,437 (94.9%)	1809 (96.8%)	266 (95.0%)
Yes	1268 (5.1%)	59 (3.2%)	14 (5.0%)
Place of injury ^g^			
Home/usual residence	14,949 (62.0%)	1385 (75.6%)	207 (78.1%)
Other place	9153 (38.0%)	446 (24.4%)	58 (21.9%)
Injury intent ^h^			
Unintentional injury	23306 (94.4%)	1799 (96.5%)	260 (93.5%)
Other intent	1395 (5.6%)	66 (3.5%)	18 (6.5%)
Burn wound management in theatre ^i^			
No	6325 (25.7%)	520 (28.0%)	57 (20.7%)
Yes	18277 (74.3%)	1339 (72.0%)	219 (79.3%)
LOS, median (IQR) days ^j^	4.0 (1.6, 9.8)	4.0 (1.5, 9.1)	4.6 (2.6, 11.7)
Discharge disposition ^k^			
Home/usual residence	21,371 (86.4%)	1674 (89.7%)	246 (87.9%)
Other disposition	3358 (13.6%)	193 (10.3%)	34 (12.1%)

Data presented as frequency (percentage) unless otherwise specified. BRANZ = Burns Registry of Australia and New Zealand; IQR = interquartile range; LOS = length of stay; TBSA = total body surface area. Data missing for ^a^ 4 admissions, ^b^ 10 admissions, ^c^ 32 admissions, ^d^ 614 admissions, ^e^ 349 admissions, ^f^ 72 admissions, ^g^ 727 admissions, ^h^ 81 admissions, ^i^ 188 admissions, ^j^ 17 admissions, and ^k^ 49 admissions.

## Data Availability

Access to BRANZ data requires ethics approval from an institutional human research ethics committee. Further information about requesting data from the BRANZ is available online: https://www.monash.edu/medicine/sphpm/branz/data-requests.

## Supplementary Materials

The following supporting information can be downloaded at: https://www.mdpi.com/article/10.3390/ijerph19159578/s1, Figure S1: Case distribution by country and time of injury; Figure S2: Age group distribution by age and country. M = Month; Y = Years.
