# Supplementary material for: Comparing Peak Burn Injury Times and Characteristics in Australia and New Zealand
Source: Int J Environ Res Public Health. 2022 Aug 4;19(15):9578. doi: 10.3390/ijerph19159578 (PMC9368485; doi:10.3390/ijerph19159578)
Supplement: Supplementary file 1 [file ijerph-19-09578-s001.zip › ijerph-1793452-supplementary.pdf]

Supplementary Materials

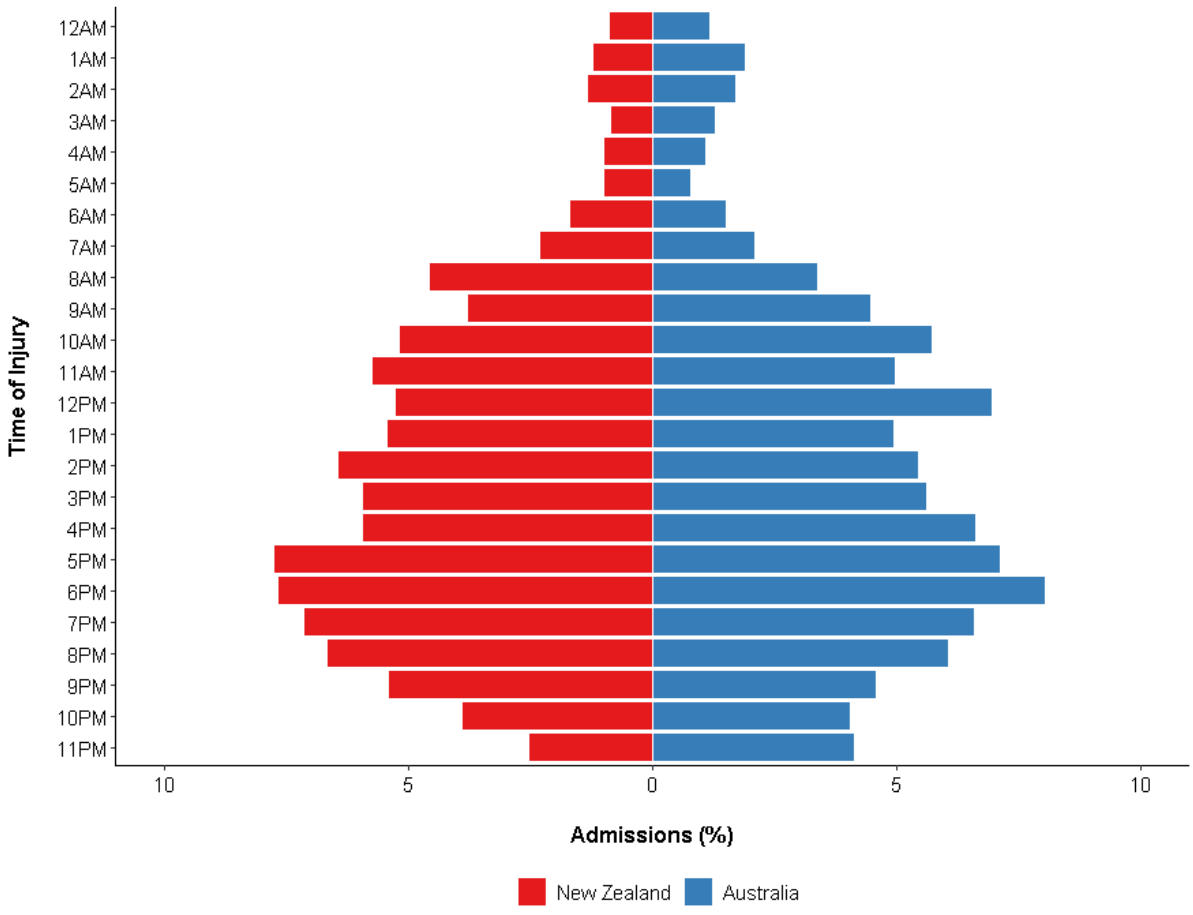

Figure S1. Case distribution by country and time of injury.

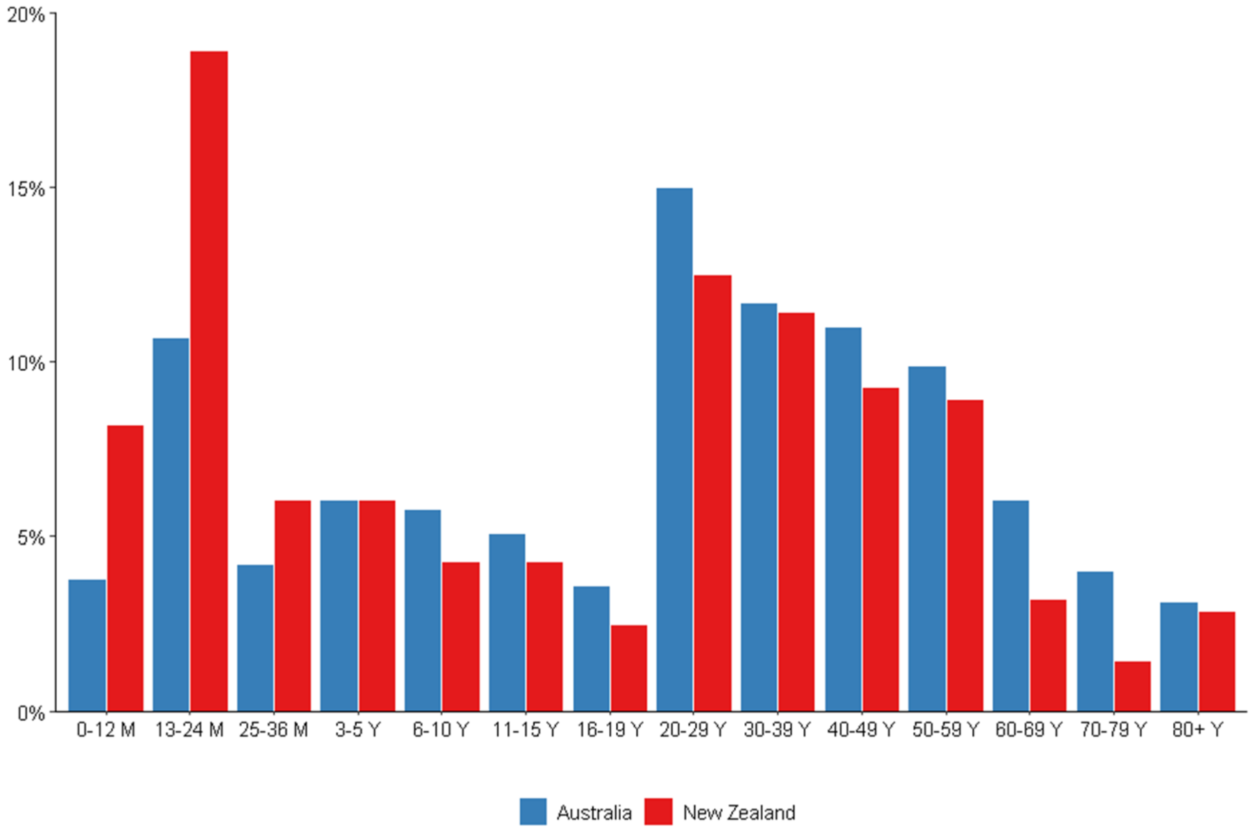

Figure S2. Age group distribution by age and country. M = Month; Y = Years.
